# Medical care for spinal diseases during the COVID-19 pandemic

**DOI:** 10.6061/clinics/2020/e1954

**Published:** 2020-04-30

**Authors:** Ricardo Teixeira e Silva, Alexandre Fogaça Cristante, Raphael Martus Marcon, Tarcísio Eloy Pessoa de Barros-Filho

**Affiliations:** Instituto de Ortopedia e Traumatologia (IOT), Hospital das Clinicas HCFMUSP, Faculdade de Medicina, Universidade de Sao Paulo, Sao Paulo, SP, BR

Coronavirus disease (COVID-19), caused by the novel coronavirus (SARS-CoV-2), reached pandemic levels in March 2020, and has the potential to overburden health systems, compromise medical staff, and deplete essential hospital supplies. To minimize these adverse consequences and guarantee adequate treatment for patients with other conditions, it is necessary to develop balanced strategies 1. Healthcare professionals must be prepared for abrupt changes in the availability of hospital beds and human and material resources during this period, and must be aware that hospitals can become locations of elevated transmission of the disease 2.

Outpatient care should be restricted and performed only in cases of great need. The Brazilian Ministry of Health has temporarily authorized and regulated telemedicine activity in the country during the pandemic period. This decision may reduce the circulation of people and must be considered by doctors and health care services to preserve the health of patients and avoid overcrowding in health units 3.

Elective surgeries should be considered on a case-by-case basis, and postponed if possible. This action reduces unnecessary foot traffic in hospitals, reduces the spread of the disease, conserves hospital supplies, and preserves the health of surgical teams 2. Nevertheless, it is essential that health services develop strategies for performing urgent and emergency surgeries during the crisis period.

To guide specialists involved in the treatment of spinal disorders, this editorial summarizes the recommendations of the North American Spine Society 4, the American College of Surgeons 5, and the document signed by the Brazilian Spine Society, the Brazilian Society of Neurosurgery, and the Brazilian Society of Orthopedics and Traumatology 6 (Table 1).

The characteristics of the COVID-19 pandemic are dynamic and distinct between regions and even between hospitals, therefore, therapeutic decisions must be individualized, and discussed with patients. The clinical condition of the patient and the availability of medical staff, personal protective equipment (PPE), and intensive care unit beds should be considered. Surgeons must also assess the risk of transmission of the virus in each region and in specific intrahospital sectors 4.

## GENERAL CONSIDERATIONS FOR SURGICAL ASSISTANCE DURING THE COVID-19 PANDEMIC

It is essential that professionals who provide medical assistance during the pandemic receive adequate training to avoid the infection of patients, companions, and the medical team itself. Strategies for reducing the length of hospital stays and limiting the number of visitors should be encouraged, as well as the isolation of patients with COVID-19 in inpatient units and in operating rooms (ORs) 2. Handwashing and hand hygiene with alcohol-based solutions should be encouraged whenever necessary, and the use of disposable resources should be prioritized 1.

Health care professionals should constantly assess for respiratory symptoms, and patients with suspected or confirmed COVID-19 should be identified. Such patients must receive appropriate clinical evaluation to analyze the risks and benefits of the procedure and the most appropriate time to perform it 2. Lei et al. suggest that surgical procedures in patients infected with SARS-CoV-2, even if they are asymptomatic, are implicated in the development of more severe forms of the disease and a higher mortality rate 7.

The following are recommended for surgical procedures with patients who are suspected or confirmed cases of COVID-19: minimally invasive procedures with shorter operation times, careful and proper anesthetic planning to accelerate intubation and reduce manual ventilation, preference for the prone position to prevent droplet spread, minimization of the use and power of electrocautery, and avoidance of contamination of people and external materials with body fluids 1,8.

Regarding the OR, the number of professionals involved in the surgical procedure and the circulation of people should be limited as much as possible. Ideally, everyone in the OR should use the following types of PPE: masks (n95/PFF2), long sleeve disposable coats, protective goggles or face shields, disposable caps, shoe covers, and disposable double pairs of gloves. Special attention must be given to donning and doffing procedures, as proper procedures are often neglected 1,8.

Efforts must be made to reduce contact and proximity between patients and health professionals. Strategies to accomplish this include anesthetic recovery in the OR, short and planned intrahospital routes, restrictions on the number of professionals responsible for the internal transport of patients, and the avoidance of transfers between different hospital sectors. The use of disposable masks by patients is recommended during each of these steps 1,9.

Strict techniques for the proper disposal of contaminated materials, the sanitization of ORs and surgical materials, and the maintenance of adequate ventilation systems must also be developed in cooperation with the internal hospital infection commission and clinical engineers 1,2.

In summary, the COVID-19 pandemic is especially challenging due to the dual efforts to control the pandemic and simultaneously guarantee essential healthcare. The recommendations presented in this editorial may help spine surgeons to make decisions and should be analyzed carefully according to the evolution of the pandemic and the concerns of each region and hospital.

## AUTHOR CONTRIBUTIONS

All authors participated equally in the realization of this work, both in the search for articles and in the writing and revision of the text.

## Figures and Tables

**Table 1:** Spine surgery recommendations during the COVID-19 pandemic.

Category	Clinical Considerations	Recommendation
Emergent	• Progressive or severe neurologic deficit due to neurologic compression from any cause (*e.g.*, infection, tumor, fracture, disc herniation, cauda equine syndrome) • Spinal instability at risk of causing neurologic injury from any cause (*e.g.*, fracture types B or C according to AO Spine Classification, tumor, infection) • Sepsis secondary to epidural abscess or spondylodiscitis • Epidural hematoma • Postoperative wound infection • Cerebrospinal fluid leakage	- Do not postpone the procedure/treatment. - The surgical procedure must be performed within 24-48 hours. If this is not possible, the patient must be transferred to another center.
Urgent	• Refractory painful radiculopathies (secondary to conditions such as disc herniation, spinal stenosis, spondylolisthesis) requiring hospitalization • Acute cervical or thoracic myelopathy • Spine fractures without instability but with surgical indication (fracture types A3 and A4) • Pathological fractures with instability (SINS > 12) but without neurological deficit • Wound dehiscence	- If the conditions are favorable, the surgical procedure must be performed within 3-7 days. - If this is not possible, the patient must be transferred to another center.
Elective	• Degenerative spinal disorders such as degenerative disc disease, some disc herniations, spinal stenosis, or spondylolisthesis, without significant neurologic deficit • Hardware failure/pseudoarthrosis without neurological deficit or critical instability • Scoliosis and/or kyphosis correction	- Consider postponing the procedure/treatment until the pandemic is under control.
